# Development of a national proficiency test for SARS-CoV-2 detection by PCR in Colombia

**DOI:** 10.7189/jogh.13.06029

**Published:** 2023-10-13

**Authors:** Sergio L Dávila, John E Leguizamón, Andrés F León, Katherin Holguín, Esther C Barros, Sergio Y Gomez

**Affiliations:** 1 Universidad Nacional de Colombia, Department of Chemistry, Faculty of Science, Bogotá, Colombia; 2 Instituto Nacional de Metrología, Bioanalysis Working Group, Bogotá, Colombia; 3 Instituto Nacional de Salud, Virology department, Bogotá, Colombia

## Abstract

**Background:**

Proficiency testing (PT) is a tool for ensuring the validity of results of testing laboratories and is essential when laboratories are working with assays authorised for emergency use or implementing novel techniques for detecting emerging pathogens.

**Methods:**

In collaboration with the National Health Institute of Colombia and with international support, we developed a qualitative PT for severe acute respiratory syndrome coronavirus 2 (SARS-CoV-2) by reverse transcription polymerase chain reaction (RT-PCR). A proficiency test item (PTI) based on reference material (research grade) produced by the National Institute of Standards and Technologies (NIST) was prepared and characterised using three positive samples with varying concentrations of SARS-CoV-2 ribonucleic acid (RNA) and two negative (control) samples. Tests were distributed to 121 laboratories across the national network of public health laboratories in Colombia.

**Results:**

Positive samples had varying concentrations of SARS-CoV-2 RNA and were quantified by digital PCR (RT-ddPCR) assays for the E gene of SARS-CoV-2. We tested the ability of laboratories to detect low and high levels of viral RNA using samples with SARS-CoV-2 RNA concentrations of 1417 ± 216, 146 ± 28, and 14 ± 10 copies /uL (expanded uncertainty, k = 2, 95% confidence level) We also performed a semiquantitative analysis of instrumental responses (Ct values) reported by participating laboratories and homogeneity, stability, and characterisation studies of the produced materials to determine the adequacy of these materials and methods for use in the qualitative PT scheme. The PT evaluated reports for individual target genes from each laboratory; 98.3% of laboratories had satisfactory performance and the remaining 1.7% of laboratories had unsatisfactory performance for the detection of at least one of the reported genes.

**Conclusions:**

This PT scheme identified the potential metrological weaknesses of laboratories in the detection of SARS-CoV-2 by RT-PCR and may facilitate improvements in the quality of measurements from the perspective of public health surveillance.

The spread of the recently discovered severe acute respiratory syndrome coronavirus 2 (SARS-CoV-2) led to the declaration of coronavirus 2019 (COVID-19) pandemic by the World Health Organization (WHO) on 11 March 2020. The COVID-19 outbreak began in China in late December 2019. To date, more than 660 million cases and 6.6 million deaths have been confirmed worldwide [1]

Almost immediately, several reverse transcription quantitative real-time polymerase chain reaction (RT-qPCR) detection protocols were created based on the SARS-CoV-2 genomic RNA sequence and published in several journals and on the WHO website [2-6]. Simultaneously, several in vitro diagnostic (IVD) SARS-CoV-2 detection tests were developed based on these protocols under the Food and Drug Administration’s Emergency Use Authorization and the WHO’s Emergency Use Listing, among others, in an attempt to detect and identify positive and negative COVID-19 cases. The detection of the SARS-CoV-2 virus was a part of the primary response in the management and control of the disease.

Countries worldwide implemented contingency measures to control the outbreak, such as the establishment of national laboratory networks for the detection of SARS-CoV-2. After national sanitary organisation approval, laboratories began using the WHO protocols and available IVD devices. Although each detection kit has its own positive and negative controls, not all protocols have the same performance characteristics. Accordingly, the use of reference materials (RM) as internal or external quality controls [7] and participation in proficiency tests (PT) [8] play key roles in ensuring reliable results. During the COVID-19 pandemic, RM, reagents, and consumables for molecular biology were not widely available, and the transfer of biological samples between countries was restricted by national biosafety protocols.

To strengthen the national network of public health laboratories responsible for SARS-CoV-2 detection in Colombia, the National Institute of Health (INS) and the National Metrology Institute (INM), with international support from the National Institute of Standards and Technology (NIST) and the United Nations Industrial Development Organization (UNIDO), developed a qualitative PT to evaluate the performance of authorised laboratories in detecting SARS-CoV-2 using RT-qPCR. Accordingly, a proficiency test item (PTI) based on NIST reference material was developed and characterised across a range of viral concentrations and distributed to national laboratories. Each laboratory then evaluated the PTI using their implemented protocols. The technical performance of each laboratory was evaluated for each target sequence reported based on the reported results. As the PTI was a ribonucleic acid (RNA) solution, the PT did not cover the RNA extraction process.

## METHODS

### Production of the proficiency test item

We used RGTM10169 NIST material, a two-vial solution containing synthetic RNA fragments from the SARS-CoV-2 genome with a nominal concentration of 5 × 10^6^ copies/μL in a background of 5 ng/μL human Jurkat RNA [9], as an RNA template (Figure S1.1. in the **Online Supplementary Document**). Solutions were mixed and diluted in 1 mM citrate buffer pH 6.4 (Invitrogen, AM 7001) to obtain a working solution with a concentration of 1 × 10^5^ copies/uL.

The primers and probes (Biosearch Technologies, Petaluma, CA, United States, purified by HPLC) intended for qPCR and dPCR method validation covered the E, N, and RdRp viral regions (Table S1.1. in the **Online Supplementary Document**). We used the RNAaseP gene as internal control for human RNA.

### RT-PCR methods

One-step reverse transcription quantitative real-time and digital PCR methods (previously validated) were used for PTI characterisation. Quantitative real-time PCR (qPCR) was performed using a CFX 96 Deep Well Thermocycler (BioRad cat. 1855196). Each 11 μL reaction mixture was prepared using one iTaq Universal Probes One-Step kit (BioRad cat 1725141) with 1 U iScript Reverse Transcription Supermix (BioRad Cat 1708841), 600 nM forward primer, 800 nM reverse primer, 300 nM probe, 2 μL template (1x Tris-EDTA buffer was used as a non-template control), and nuclease-free water. The amplification cycle consisted of a reverse transcription phase at 50°C for 10 minutes, polymerase activation at 95°C for one minute, 40 cycles of denaturation at 95°C for 10 seconds and annealing-elongation at 56°C for 30 per second, and a final step at 4°C for 10 minutes with a heating ramp of 2°C per second.

### Reverse transcription digital PCR (RT-dPCR)

A QX200 platform (BioRad cat 1864001) was used for droplet digital PCR (ddPCR). The 21 μL reaction mixtures were prepared using one One-Step RT-ddPCR Advanced Kit for Probes (BioRad cat 1864021) with 20 U reverse Transcriptase, 15 mM DTT, 900 nM primers, 250 nM probes, 3 μL RNA template (1x Tris-EDTA buffer was used as a non-template control), and nuclease-free water. The amplification cycle consisted of a reverse transcription phase at 55°C for 10 minutes, polymerase activation at 95°C for three minutes, 45 cycles of denaturation at 95°C for 15 seconds, annealing-elongation at 58°C for 30 seconds, and a polymerase inactivation at 98°C for 10 minutes, and a final step at 4°C for 10 minutes with a heating ramp of 0.5°C per second.

### PTI production

Although the PT was qualitative, the PTI was quantitatively characterised to ensure its suitability for evaluating the performance of participating laboratories.

#### PTI preparation

The PTI consisted of a panel of five vials containing RNA and buffer solutions, and three positive and two negative samples. To prepare the positive samples, three different gravimetric dilutions of the NIST RGTM10169 working solution were prepared in 1 mM citrate buffer pH 6.4 (Invitrogen, AM 7001) supplemented with 0.5 ng/μL of human T-Cell Leukemia total RNA (Invitrogen, AM7858) with a nominal copy number concentration of 1000, 100, and 10 copies/μL. The two negative samples were 1 mM citrate buffer pH 6.4 with and without 0.5 ng/μL of human T-Cell Leukemia total RNA. Each sample had a volume of 80 μL and was dispensed in a 500 μL polypropylene, screw cap and seal, DNase- and RNase-free cryovial (Biologix Cat 81-7054). The five vials of each PTI were packaged in a plastic blister, plastic bag, and aluminised bag. A total of 200 panels were produced and stored at 4°C before shipping to participating laboratories.

#### PTI homogeneity and stability studies

We performed homogeneity and stability studies according to ISO Guide 35: 2017 recommendations [10]. For the homogeneity study, 15 vials per concentration were randomly selected and measured in triplicate by RT-qPCR using the E Charité assay [3].

To monitor the stability of the PTI during the PT (one month including transportation), an isochronous study was performed to evaluate the three concentration levels at 4°C using -70°C as the reference temperature. Two units were measured in triplicate by RT-qPCR using the E Charité assay every week. With some additional panels, we continued to monitor the PTI for 11 months after the PT to monitor stability. Text S3 in the **Online Supplementary Document** shows in detail the pilot production of the material, together with its performance in closed PCR platforms.

#### PTI characterisation

A copy number concentration value was assigned for each positive level of PTI from the mean of five randomly selected vials using the RT-dPCR E-Charité assay, under repeatable conditions. We calculated uncertainty per the Guide to the Expression of Uncertainty (GUM) [11], considering homogeneity (u_hom_), stability (u_sts_), and characterisation (u_char_) as principal uncertainty sources (Text S4 in **the** **Online Supplementary Document**). We also measured the PTI by by RT-qPCR using N1, N2, RdRp, and RNase P assays in triplicate for each concentration level to determine the expected laboratory responses for each sample.

### Proficiency test

Panels were shipped on gel packs in a polystyrene box according to the providers’ instructions (Biothermics S.A.) to maintain a temperature of 4 to 8°C. Delivery to all laboratories (n = 121) was completed in two days. They also received electronic forms to report the condition of the panels on receipt (temperature, physical appearance, leakages, and general observations) and report measurement results and technical information (platform, commercial assays, genomic targets, volumes, amplification conditions, and Ct/Cq results, among others).

#### Performance evaluations and statistical analyses

We based performance evaluations on the number of samples correctly reported according to ISO 13528:2015, numeral 11 [12]. We considered performance “satisfactory” if the laboratory had ≥80% of correct results and “not satisfactory” if this proportion was <80%. As the participating laboratories reported results from a different number of tests and molecular RNA targets, individual performance evaluations were conducted for each gene or assay used by each laboratory.

The instrumental response values (Ct/Cp/Cq) reported by some laboratories did not affect their evaluation; however, they provided information on the genes and tests being used by the evaluated laboratories.

## RESULTS

### PTI homogeneity and stability study

The degree of heterogeneity calculated as relative uncertainty from the 15 vials evaluated for each positive concentration level (low, medium, and high), ranged from 0.1% to 0.3%.

**Figure 1:**
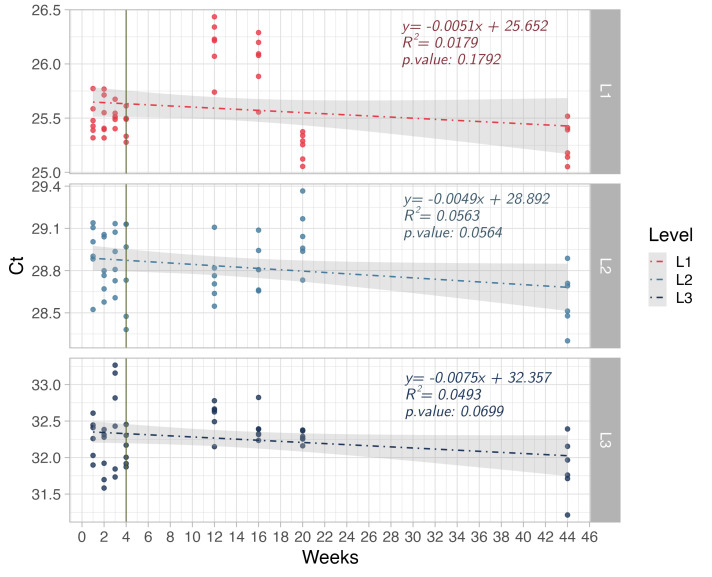
Stability analysis of PTI, L1, L2, and L3 at high, medium, and low concentrations.

Regarding stability, the regression analysis of the positive samples demonstrated that the slopes were not significantly different from zero (Text S5 and Table S5.1. in the **Online Supplementary Document**). According to this analysis, we detected no instability in the material at the three concentrations evaluated at 4°C during the four weeks. After the PT, we monitored the PTI for a further 11 months to measure the degree of instability over time. The regression analysis showed no significant association between sample concentration and time (**Figure 1**).

The contribution of instability to relative uncertainty values was calculated from the standard deviation of the regression data (Table S4 in the **Online Supplementary Document**). After 11 months, we observed relative uncertainties for the high (0.4%), medium (0.6%), and low concentration samples (1.0%). Vertical line represents the break between the short-term (four weeks) and long-term stability studies.

### PTI characterisation

We used one-factor analysis of variance (ANOVA) to determine the actual concentration of each positive sample. We calculated the combined standard uncertainty of the assigned value (u_RM_) from the combination of the uncertainty values from the characterisation (u_char_), the homogeneity (u_hom_), and stability studies (u_stab_) (**Table 1**, Text S5 and Tables S5.2.-S5.4. in the **Online Supplementary Document**).

**Table 1:** Reference value and relative uncertainty for the PTI*

Level	Assigned value in copies/μL	u_char_	u_hom_	u_stab_	u_RM_	U_RM_†
Low	14.6	27.9	0.3	0.5	28.8	60.5
Medium	146	7.2	0.1	0.4	7.5	15.7
High	1417	3.9	0.2	0.6	4.5	9.4

u_char_ – characterisation uncertainty, u_hom_ – homogeneity uncertainty, u_stab_ – stability uncertainty, u_RM_ – standard uncertainty assigned to the reference material, k – coverage factor

*Values presented as % unless otherwise specified

†U = expanded uncertainty, k = 2, 95% confidence level.

Regarding the expected instrumental response (Ct values) for the PTI using different target assays, **Table 2** shows the results for N1, N2, RdRp, and RNaseP measurements using previously validated assays. For RNaseP, we performed measurements in the three positive samples and the negative sample which included human RNA.

**Table 2:** Ct values according to sample concentration and target sequence used

Level	N1	N2	RdRp	RNaseP
Low	26.31	25.20	26.95	27.39
Medium	29.55	28.32	30.30	27.38
High	32.78	32.10	33.37	27.20
Negative-1	-	-	-	27.26

### Proficiency Test

#### Laboratories participating in the PT

A total of 124 laboratories were enrolled in the PT, with 121 reporting results. Laboratories were either public (33%) or private (67%) organisations and included medical laboratories (41%), hospitals (18%), universities (23%), public health laboratories that support disease surveillance (12%), investigation centres (5%), and reference laboratories (3%).

#### SARS-CoV-2 RT-qPCR test in the PT

**Figure 2:**
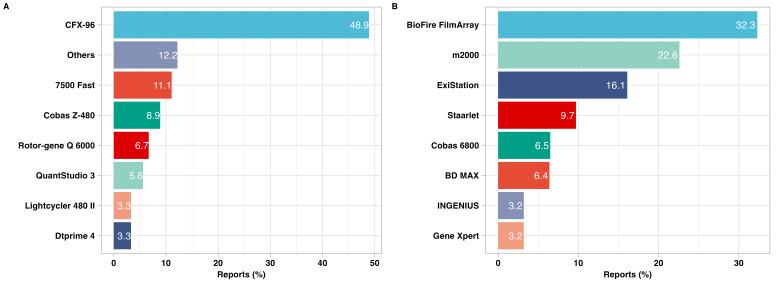
Distribution of platforms used by laboratories. **Panel A.** Open platforms. **Panel B.** Closed platforms.

Overall, 74.4% of participating laboratories used open measurement platforms, which only performed reverse transcription and amplification reaction of samples; the sample input volumes varied according to the commercial or implemented protocols used. The remaining 25.6% of laboratories used closed platforms, with most laboratories preparing dilutions of each sample to a final volume of 900 μL (**Figure 2**, Text S6 and Table S6.1. in the **Online Supplementary Document**). The volumes required to achieve the required concentrations for individual platforms (ranging from 250 μL to 900 μL) were taken from the prepared dilutions.

**Figure 3:**
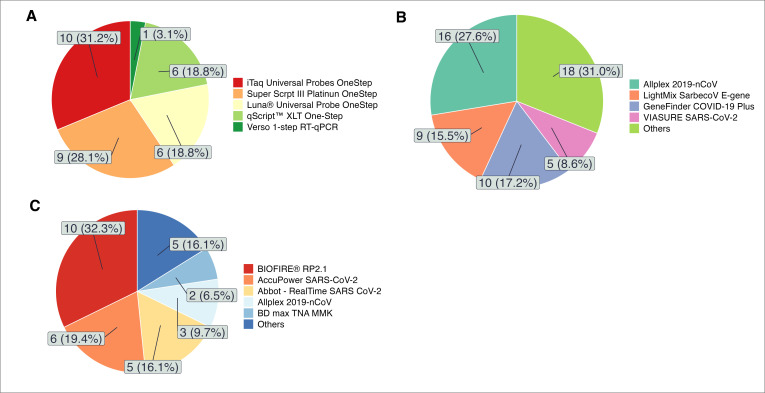
Distributions of RT-PCR kits used by laboratories. **Panel A.** In-house protocols. **Panel B.** Commercial kits open platforms. **Panel C.** Commercial kits closed platforms.

For SARS-CoV-2 detection, all laboratories (using open and closed platforms) performed RT-PCR in a single step. The RT-qPCR kits used were classified into three categories (Figure 3, Text S6 and Table S6.1. in the **Online Supplementary Document**):

1. In-house protocols: those composed of DNA polymerase, RNA retrotranscripase, buffer, nucleotides, and cofactors. The primers and probes were purchased separately and were selected by the laboratory, typically from protocols reported by the WHO. This type of kit was used by 25% of participating laboratories;
2. Commercial kits for the detection of SARS-CoV-2 using open platforms. This type of kit was used by 51% of participating laboratories;
3. Commercial kits for the detection of SARS-CoV-2 using closed platforms. This type of kit was used by 24% of participating laboratories.

**Figure 4:**
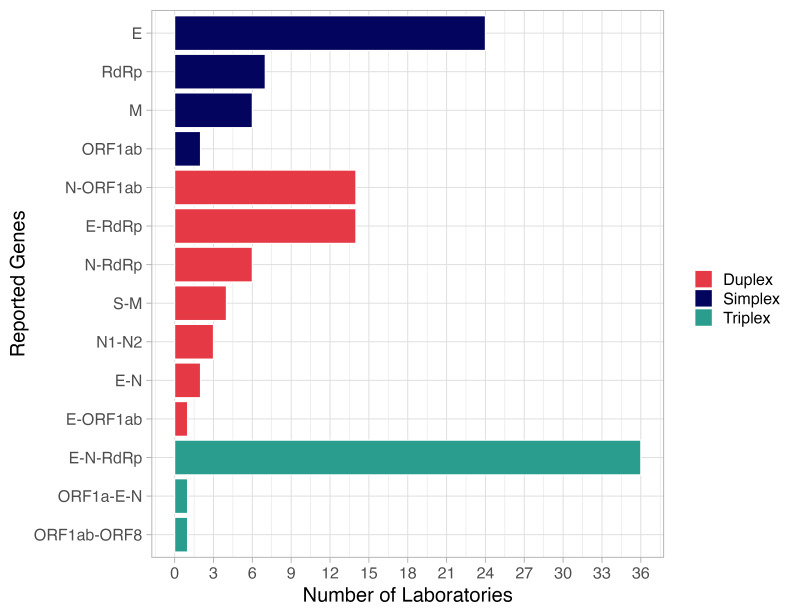
Number of RNA targets tested for by participating laboratories.

Regarding RNA targets, 32.2% of laboratories tested for a single gene, most frequently for the E gene, followed by the RdRp region and the M gene; 36.4% of laboratories tested for two genes using duplex assays, with E-RdRp and N-ORF1ab being the most frequently tested genes. The remaining 31.4% of laboratories tested for three genes, with the E-N-RdRp triplex assay the predominant test performed (**Figure 4**).

The human RNase P gene, used as an internal control in most commercial assays, was reported for by only 17 laboratories. However, performance evaluations were performed based on the detection of viral genes only.

#### Limit of detection values and PTI volumes reported by laboratories

Laboratories reported various values and units for limit of detection (including copies/μL, copies/mL, copies/reaction, genomic equivalents/mL, μM, ng/μL, and Ct values). The volumes of the PTI used by participating laboratories using open PCR platforms ranged from 2 to 10 μL per RT-PCR reaction, approximately equivalent to 28 to 140 copies per RT-PCR reaction (according to the assigned values for the PTI) (Text S8, Table S8.1-S8.2., and Figure S8.1 in the **Online Supplementary Document**)

#### Performance evaluations

**Figure 5:**
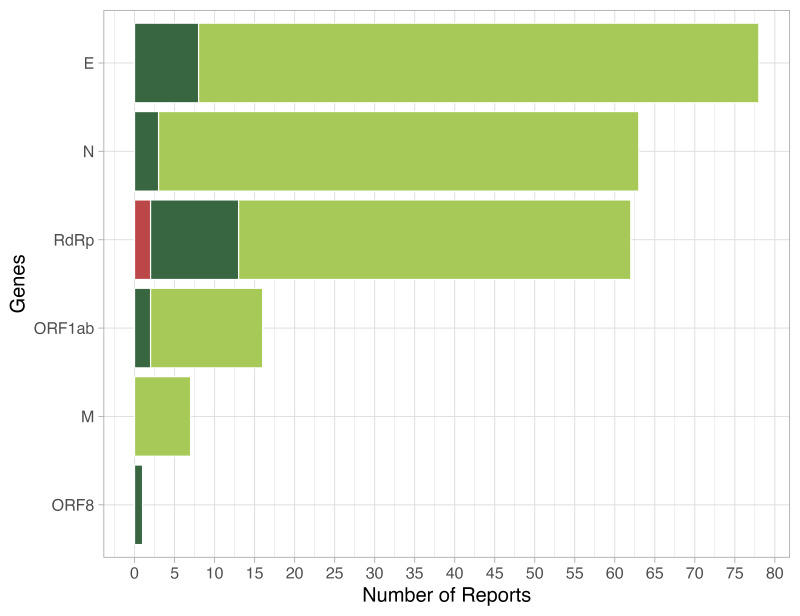
Causes of error in the PT.

We performed performance evaluations of the SARS-CoV-2 PT for each reported assay for each participating laboratory according to the selected evaluation criteria. Accordingly, 98.3% (n = 119) laboratories had a satisfactory performance for all reported assays. The remaining 1.7% of laboratories had an unsatisfactory performance for more than two samples with at least one of the reported assays (**Figure 5**).

**Figure 6:**
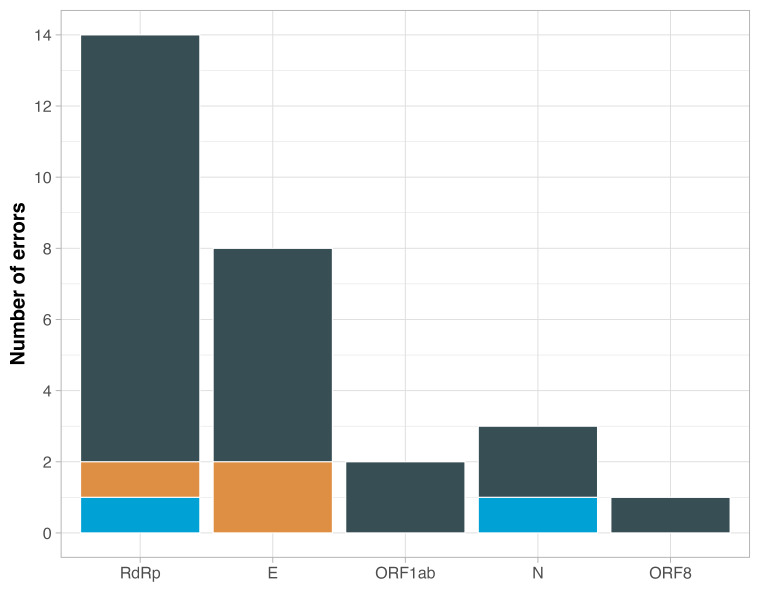
PT performance according to the assay used.

Of the laboratories with satisfactory performance, 14.3% (n = 17) failed to correctly measure one of the samples contained in the panel using one or more assays. The most frequent cause of error was the incorrect classification of the positive sample (false negative) with the lowest concentration (14 copies/μL), with a frequency of 75%, 75%, and 67% with the use of the RdRp, E, and N gene assays, respectively (**Figure 6**); other errors occurred due to false positives (positive classification of a negative sample) and unreported results.

#### Semiquantitative results according to Ct values

**Figure 7:**
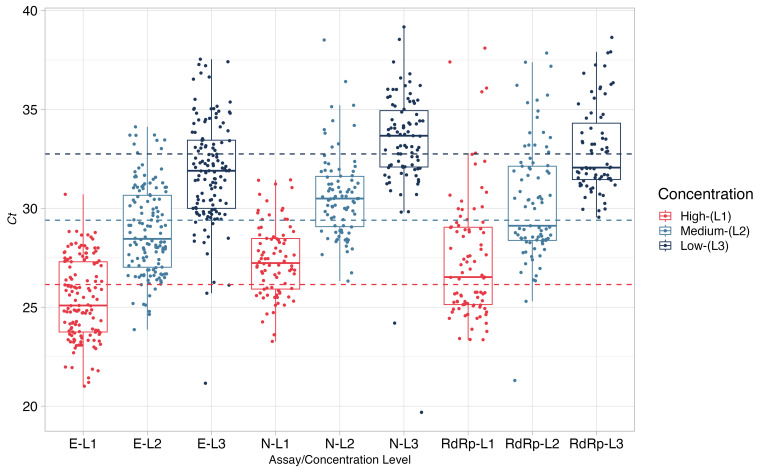
Semi-qualitative analysis demonstrating the distribution of Ct values from assays for the E, N, and RdRp genes using open platforms.

The RT-qPCR instrumental responses (Ct, Cp, and Cq) reported by the laboratories were not used in the performance evaluation. **Figure 7** shows the distribution of the Ct values obtained for the E, N, and RdRp genes from positive samples at different concentrations.

## DISCUSSION

While the PT was a qualitative exercise, the PTI was produced and characterised quantitatively to challenge laboratories in detecting medium to low copy numbers of SARS-CoV-2 RNA in the provided samples, which is above the detection limits of most in-house and commercial methods [13]. For this, a panel of three positive (with different copy number concentrations) and two negative samples (with and without human RNA) were prepared.

The homogeneity study showed that the PTI had low heterogeneity, with no changes in material stability during the PT. In the extended stability study, we observed no change in the stability of the PTI after 11 months (**Figure 1**). The maximum ranges of Ct values reported using RT-qPCR were 1.5, 1.2, and 2.5 for high, medium, and low concentrations, respectively. This indicates that material produced using the same process (including RNA source, buffers, and vials) could be used as an independent positive control for up to 11 months [14,15]. Other assays use genomic RNA, which has lower stability at low concentrations. The purification process and the size of the RNA fragment used likely have significant effects on material stability [16].

The characterisation process demonstrated that the low-, medium-, and high-concentration positive samples had a copy number concentration of 1417 ± 216, 146 ± 28, and 14 ± 10 copies/uL, respectively (where values following the  ± symbol indicate expanded uncertainty with a coverage factor k = 2 v for 95% confidence level). Although the uncertainty in these values tended to be high, particularly at low concentration levels, the material was considered adequate for the PT as the PT was qualitative (laboratories reported detection or non-detection), and as laboratories used real-time PCR (which provides Ct values) and approximately 5 to 10 μL of the provided samples, equivalent to 70 to 140 copies per reaction (which provided an amount of viral RNA that could easily be detected by most commercial and in-house RT-qPCR protocols). **Figure 7** shows the Ct values obtained from the measurement of the three positive samples, which had Ct values ranging between 20 and 40 (a common interval for qPCR measurements) [17,18]. Alongside the results of the homogeneity and stability studies (**Figure 1**), this demonstrates that the material was adequate for use in the PT, where any misclassification of positive samples would reflect the measurement method used rather than the quality of the sample provided.

In the PT, the laboratories implemented a diversity of technical tools to perform SARS-CoV-2 detection using PCR. This included the use of different commercial open and closed measurement platforms, RT-PCR kits or in-house protocols, different RNA target sequences (E, N, M, RdRp, and ORFs) with assays based on the region that encodes protein E the most reported with 78 reports, in line with CDC and WHO recommendations to use assays focused on the E, N1, and N2 genes, with subsequent confirmation using RdRp [19,20]. Accordingly, the PT evaluation was performed for each RNA target reported by each laboratory.

The participating laboratories most frequently used open measurement platforms due to economic cost, the wide range of available applications, type of reagents, and ability to modify the protocol used. On the other hand, some organisations processing a high volume of samples preferred the use of closed platforms. In both cases, the results of the PT demonstrated good performance in measuring the evaluated targets (n/N = 119/121 laboratories).

Although the RNA template did not include the S gene or the entire ORF1ab region, some laboratories reported results for these targets. The ORF1ab gene was reported in 20 different tests by five laboratories (alone or in combination with other detection targets), with these five laboratories reporting negative results for all the samples contained in the panel. As the PTI contains only a segment of the ORF1ab region (equivalent to the RdRp gene sequence), we did include these reports in the performance evaluations for the RdRp gene. Regarding reporting of the S gene, three laboratories reported results for the S gene only and four reported results for the S and M genes. Considering the detection targets used and the method of software reporting, results detecting the S gene were considered to represent satisfactory performance in detecting the M gene (which was included in the PTI). For laboratories reporting results for the M and S genes, the performance was evaluated using reports for the M gene only.

The performance of the laboratories is similar to that obtained in similar exercises carried out in other countries, varying mainly in the commercial kits and protocols used [21-24]. The most common cause of error for laboratories with satisfactory performance (i.e. with only one incorrect assignment) was a false negative report. Two laboratories with closed platforms and 12 laboratories with open platforms were unable to correctly assign the low concentration sample. Considering the use of greater volumes of this sample in the reaction mixtures (5 to 8 μL, equivalent to 70-112 copies per reaction) and considering the reported LoD values (5.6, 10, and 100 copies/reaction), all laboratories should have been able to accurately assign the low concentration positive sample. Accordingly, issues with the methods used by the laboratories may have resulted in sample misassignment, indicating these laboratories should first effectively evaluate the performance of PCR kits before their use.

Heterogeneity was observed in the LoD values and units reported by laboratories (Text S8 in the **Online Supplementary Document**), although some reported units are roughly equivalent such as copies/uL, copies/mL and genome equivalents (GE)/mL (assuming one copy per genome). However, a wide range of values were reported (from 0.004 to 20 copies/uL when transforming reported values to copies/uL), with some likely to be spurious from a technical point of view (such as 0.004 copies/uL). Other reported units, such as ng/uL and uM (if the molar weight is not available) were difficult to transform to copies/uL, thereby preventing their interpretation. Some protocols reported an instrumental response value for the LoD, such a Ct value, making transformation to copies/uL more difficult if the RM, or at least the calibration curve data, were not available. These findings reflect a lack of standardisation among IVD producers, leading to misinterpretation of results in some cases. The interpretation of the LoD allows laboratories to determine the minimum amount of viral RNA detectable and calculate type I and II error [13]

Regarding the quantitative results of open platforms, ANOVA and Tukey’s method showed a significant difference between the concentrations of the positive samples, particularly for the E gene at all concentrations (although not for the N and RdRp genes). Additionally, the gene E had the highest dispersion, and the N gene had the lowest dispersion at all three concentrations of the positive samples. Although both genes are in the same RNA fragment, this dispersion may be associated with the accessibility of primers to specific sites in the RNA template and the method optimization [25]. We observed some extreme Ct values (not shown), particularly for the N and RdRp genes, presumably given the variety of methods for a single gene, with different protocols and different amplification efficiencies, the dispersion in the data will be affected [26]. However, these reported values did not affect the overall evaluations of the reporting laboratories.

Despite these differences and considering that data were obtained from different measurement platforms, assays (commercial or in-house; simplex, duplex, or triplex), and RNA targets for each concentration of the positive sample, there is general consistency between the Ct values reported by the participating laboratories. This may represent a first step toward the standardisation of RM used for PTI.

## CONCLUSIONS

The reliable detection of SARS-CoV-2 infection is important for controlling the spread of infection and is determined by the availability of measurement assurance tools such as measurement systems, RM, inter-laboratory comparisons, and technical competence, among other factors. Further, it is important to have a common technical language that facilitates interactions between laboratories and clinicians.

Here we evaluated the competence of laboratories in detecting SARS-CoV-2 by RT-PCR. Repeatability values ranged from 1% to 21% and 1.2 to 2.3% and LoD values of 0.2 and 2.6 copies/uL were observed for RT-ddPCR and RT-qPCR, respectively, using purified samples with 0.5 to 4500 copies/uL for RT-ddPCR and 1.6 to 1.5 × 105 copies/uL for RT-qPCR.

Based on RGTM10169 NIST material and previously validated methods, a PTI was prepared and characterized at three concentrations (1417 ± 216, 146 ± 28, and 14 ± 10 copies/uL) to challenge the technical capabilities of laboratories in detecting SARS-CoV-2 RNA at medium and low concentrations.

A qualitative PT for SARS-CoV-2 detection by RT-PCR was sent to 121 laboratories across Colombia; 98.3% of the reported results were satisfactory, demonstrating that most participating laboratories have the capability to detect SARS-CoV-2 by RT-PCR. Participating laboratories used open and closed platforms with commercial and in-house protocols to detect between one and three targets, of which the E gene was the most common followed by the RdRp and N genes. Accordingly, the use of this PT may improve the quality of SARS-CoV-2 detection from the perspective of public health surveillance.

Apart from the laboratory evaluations, this exercise provided a general overview of the national inventory in terms of measurement platforms, reagents, RM, and procedures used by laboratories for SARS-CoV-2 detection. This inventory can be used to detect other pathogens of national interest and strengthen the ability of national laboratories to perform PCR measurements.

This study had some limitations. First, the PT did not include an RNA extraction phase, which is a very important step in routine measurements, as a purified RNA transcript was used. Second, it did not cover all RNA targets available for SARS-CoV-2 detection, such as the S gene and may not be suitable for the evaluation of commercial kits that detect the entire RdRp/ORF1ab gene.

Notably, our findings have relevance in the national and international context. The COVID-19 pandemic resulted in a shortage of PCR reagents due to customs restrictions on the entry of biotechnological products, which are necessary for some SARS-CoV-2 diagnostic tests. The availability of proficiency tests through national and international cooperation and technical communication between different organisations allows the coordination of efforts for detecting SARS-CoV-2. Evaluation of the competencies of testing laboratories can facilitate decision-making on strategies for managing COVID-19.

## Additional material


Online Supplementary Document
