# Supplementary material for: Development of a national proficiency test for SARS-CoV-2 detection by PCR in Colombia
Source: J Glob Health. 2023 Oct 13;13:06029. doi: 10.7189/jogh.13.06029 (PMC10569378; doi:10.7189/jogh.13.06029)
Supplement: Online Supplementary Document [file jogh-13-06029-s001.pdf]

ONLINE SUPPLEMENTARY DOCUMENT

Title: Development of a national proficiency test for SARS-CoV-2 detection by PCR in Colombia

Authors: Sergio Luis Dávila González[1]*, John Emerson Leguizamón Guerrero[2]*, Andrés Felipe León Torres[2], Katherin Holguín Agudelo[2] , Esther Cristina Barros Liñan[3], Sergio Yebrail Gomez Rangel[3]

Text S1: Starting material and PCR methods.

Reference material RGTM10169 - NIST, used in the validation of methods and to produce the Proficiency Test item, Material is composed of two 4.000 base RNA fragments covering the principal RNA targets for SARS-CoV-2 detection.

Figure S1.1 RGTM 10169 material scheme

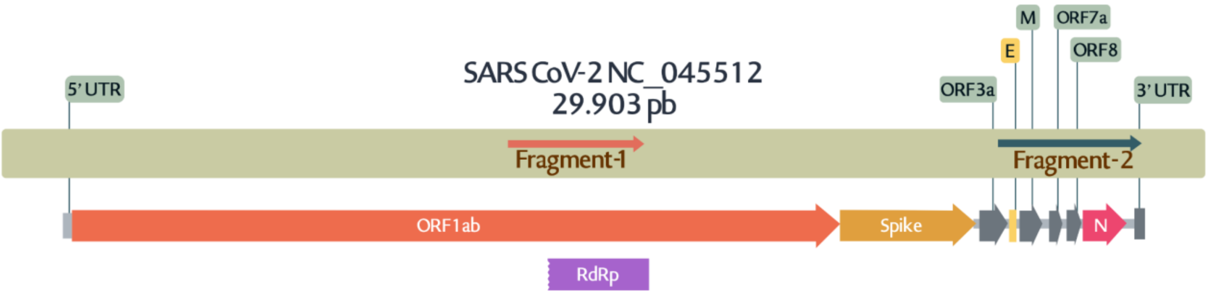

The PCR assays evaluated during method validation are shown below.

Table S1.1 Primers and probes used in this study.

Assay	Name/code	Sequence (5'-3')	Reference
E	E_Sarbeco_F	ACA GGT ACG TTA ATA GTT AAT AGC GT	[1]
	E_Sarbeco_R	ATA TTG CAG CAG TAC GCA CAC A	
	E_Sarbeco_P1	FAM-ACA CTA GCC ATC CTT ACT GCG CTT CG-BHQ	
N (China)	N-China-F	GGG GAA CTT CTC CTG CTA GAA T	[2]
	N-China-R	CAG ACA TTT TGC TCT CAA GCT G	

	N-China-P	FAM-TTG CTG CTG CTT GAC AGA TT-BHQ	
N (CDC)	2019-nCoV_N2-F	TTA CAA ACA TTG GCC GCA AA	[3]
	2019-nCoV_N2-R	GCG CGA CAT TCC GAA GAA	
	2019-nCoV_N2-P	FAM-ACA ATT TGC CCC CAG CGC TTC AG-BHQ	
RdRp	RdRp_SARSr-F	GTGAAATGGTCATGTGTGGCGG	[1]
	RdRp_SARSr-R	CAAATGTTAAAAACACTATTAGCATA	
	RdRp_SARSr-P2	HEX-CAGGTGGAACCTCATCAGGAGATGC-BHQ	
RNasaP	RP-F	AGA TTT GGA CCT GCG AGC G	[3]
	RP-R	GAG CGG CTG TCT CCA CAA GT	
	RP-P	HEX- TTC TGA CCT GAA GGC TCT GCG CG-BHQ	

Text S2: Method Validation

Once the RT-PCR methods had been optimized (PCR efficiency, annealing temperature, reaction time, and heating ramp), the selected performance parameters were evaluated for each target gene (Table S2). The working interval for each PCR method was evaluated by linear regression analysis using ordinary least squares between the working solution dilutions versus the instrumental response (for qPCR) and the concentration response (for dPCR) in the selected ranges. The acceptance criteria was a slope statistically different from zero and a correlation coefficient >0.99. Precision was evaluated using analysis of variance with the inter-day difference used as the variation factor. A relative standard deviation of <25% was set as the acceptance criteria. The LoQ was defined as the minimum concentration level of the working interval.

qPCR validation

Serial 10-fold gravimetric dilutions prepared from the working solution were at concentrations ranging from 0.2 to 200,000 copies/µL, To determine the LoD of RT-qPCR, serial gravimetric dilutions from 6 to

0.2 copies/μL were measured in four replicates. The LoD was defined as the minimum concentration where four replicates were positive.

dPCR validation

Gravimetric dilutions were prepared from the working solution at a range of 4500 to 0.2 copies/μL to evaluate the performance selected parameters (linearity, repeatability, inter-day intermediate precision, LoD, and LoQ), For RT-dPCR, serial gravimetric dilutions from 0.5 to 0.04 copies/μL were used in triplicate. The LoD was defined as the lowest concentration with a minimum of 9 positive partitions in three replicates [11]. Table S2 summarizes the results for each validation parameter.

Table S2. Summarized results for RT-PCR validation methods.

Parameter	Observation	RT-ddPCR	RT-qPCR
Linearity	Linear interval (copies/μL)	0.5–4500	$1.60–1.51 \times 10^5$ [a]
Precision	Repeatability (%)	1.1(high)–20.9 (low)[b]	1.2–2.3 [b]
	Intermediate precision (%)	0.7–12	4.4–6.3[b]
LoD	Maximum estimated concentration (copies/μL)	0.2	2.6
	Copies/reaction	5	29

[a] lowest concentration corresponding to the linear range for the N assay.

[b] Range of maximum precision values in terms of repeatability for the highest and lowest concentrations in the linear range corresponding to the N (CDC) and E INM assays. Intermediate precision values corresponds to the RdRp assay only.”

Regarding the validation of PCR methods required for PTI characterization, ddPCR, has a higher dispersion for low concentration samples, and has a lower LoD compared to the qPCR method. Specificity has previously been evaluated using the reference methods (Table S1). The repeatability of the ddPCR method at low concentrations contributed to higher uncertainty during PTI characterization. Both qPCR and ddPCR methods have satisfactory performance in measuring SARS-CoV-2 RNA copy number (copies/µL) in samples containing 10 to 1000 copies/uL (Table S2). Defined concentrations for reference material.

Text S3: Pilot Study: Proficiency Test Item

Prior to PTI production, a pilot study was performed as a prospective study to evaluate the concentration levels, homogeneity, and stability of the materials at −20°C and 4°C, with −70°C used as a reference temperature (Figure S2).

Figure S3.1 Short-term stability results for pilot study evaluating three concentration levels at 4°C and −20°C, reference temperature (yellow dots) (−70°C).

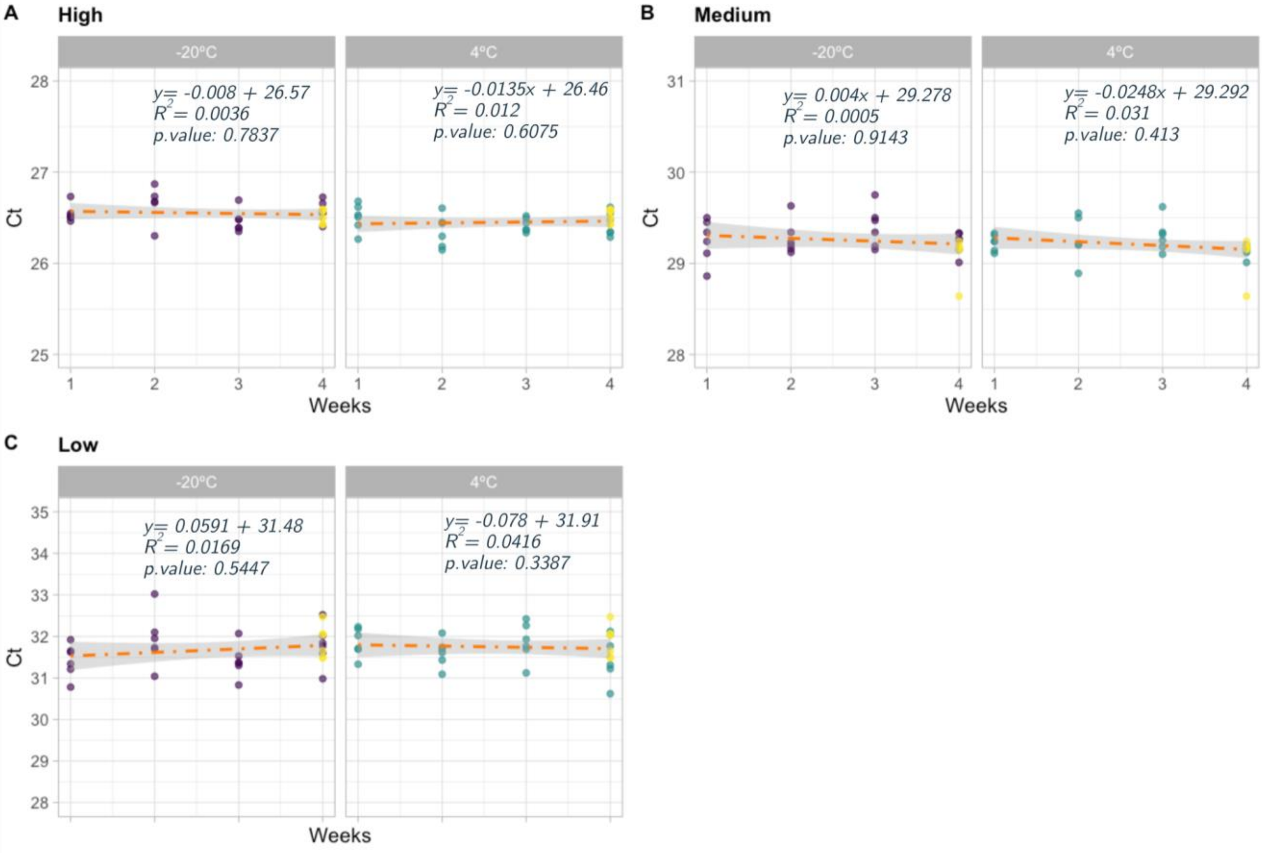

Additionally, as some laboratories performed PT with closed platforms (automated RNA extraction, purification, and RT-PCR), three dilutions were prepared from a 10 copies/µL SARS-CoV-2 RNA solution. Aliquots containing 20, 40, or 60 µL of the 10 copies/µL SARS-CoV-2 RNA solution were diluted with 1 mM citrate buffer pH 6.4 (Invitrogen, AM 7001) to a final volume of 300 µL. Samples were measured using two independent platforms, Filmarray (BioFire®) and Cobas® (Roche). The Cobas® platform required a total volume of 650 µL.”

Additionally, as some laboratories performed PT with closed platforms (automated RNA extraction, purification, and RT-PCR), three dilutions were prepared from a 10 copies/µL SARS-CoV-2 RNA solution. Aliquots containing 20, 40, or 60 µL of the 10 copies/µL SARS-CoV-2 RNA solution were diluted with 1 mM citrate buffer pH 6.4 (Invitrogen, AM 7001) to a final volume of 300 µL. Samples were measured using two independent platforms, Filmarray (BioFire®) and Cobas® (Roche). The Cobas® platform required a total volume of 650 µL.

The results of the pilot study determined the PTI preparation conditions (dilutions, volumes, nominal concentrations, buffers, and vial materials) required to ensure the proposed PTI was suitable for open and closed platforms (Table S3).

Table S3.1 Results from the pilot study of materials used in closed platforms.

Dilution	Total, copies in 300 µL	Platform		
		BioFire ® FilmArray® COVID-19 TEST v1.0	BioFire ® FimArray® Respiratory Panel 2.1	Cobas® 6800 SARS-CoV-2 Test
1	200	Detected	Detected	Positive (35.13, 36.42) *
2	400	Detected	Detected	Positive (34.5, 35.29)
3	600	Detected	Detected	Positive (36.22, 37.41)

*Results in parentheses correspond to CT values for ORF1ab and E genes.

Based on the results of this study, a reference material for the PTI was developed. Homogeneity, stability, and characterization studies were performed to demonstrate the PTI was fit for purpose.

Text S4: Reference Material characterization

Homogeneity study

The heterogeneity of the material was evaluated using one-factor ANOVA. The uncertainty component for homogeneity (u_{hom}) was calculated according to Eq 1.

$$u_{hom} = u_{bb} = s_{bb} = \sqrt{\frac{MS_{between} - MS_{within}}{n}} \qquad Eq\ 1$$

where u_{bb} is the uncertainty associated with between-unit variability that corresponds to s_{bb} (the standard deviation of between-unit component from a homogeneity study), $MS_{between}$ is the between-group mean square, MS_{within} is the within-group mean square or repeatability variance, and n is the number of replicates.

When MS_{within} is greater than $MS_{between}$, the s_{bb} was calculated according to Eq 2. [9]

$$s_{bb} = \sqrt{\frac{MS_{within}}{n}} * \sqrt[4]{\frac{2}{v_{MS_{within}}}} \qquad Eq.2$$

Where $v_{MS_{within}}$ are the degrees of freedom of the MS_{within}.

Stability study

The uncertainty contribution due to stability was calculated from regression analysis as the slope standard deviation and the time in weeks (Eq 3). The relative uncertainties were calculated using the concentration value as a reference.

$$u_{lts} = s(b) * t \qquad Eq\ 3$$

Value assignment

The previously validated RT-dPCR E-Charité assay was used under repeatable conditions in triplicates to determine the mean of five selected vials per concentration level (x_{char}); where the components associated with homogeneity (δ_{hom}) and stability (δ_{stab} were zero (Eq 4).

$$X_{RM} = x_{char} + \delta_{hom} + \delta_{stab} \qquad Eq\ 4$$

The combined standard uncertainty of the reference value was calculated according to Eq 5,

$$u_{RM} = \sqrt{u_{char}^2 + u_{hom}^2 + u_{stab}^2} \qquad Eq\ 5$$

Where u_{char} comes from the mathematical model that describes the copy number concentration measured (C_{PTI}) combined with the measurement precision (R; Eq 6 and 7)

$$C_{PTI} = \frac{\lambda}{V*d} * R \qquad Eq\ 6$$

$$u_{C_{PTI}} = C_{PTI} * \sqrt{\left(\frac{u_\lambda}{\lambda}\right)^2 + \left(\frac{u_V}{V}\right)^2 + \left(\frac{u_d}{d}\right)^2 + \left(\frac{u_R}{R}\right)^2} \qquad Eq\ 7$$

Text S5: Reference Material characterization results

Table S5.1. Regression analysis for short- and long-term stability study for PTI

Long-term Stability–Regression values						
Concentration	Coefficients	Estimate	Std. Error	t-value	R^2	p-value
High	Intercept	25.6532	0.070004	366.454	0.03887	0.1792
	Slope	-0.005135	0.003765	-1.364		
Medium	Intercept	28.9823	0.04556	634.081	0.07588	0.0564
	Slope	-0.00479	0.002451	-1.957		
Low	Intercept	32.35755	0.075663	457.656	0.06962	0.06997
	Slope	-0.007549	0.004069	-1.855		
Short-term Stability–Regression values						
Concentration	Coefficients	Estimate	Std. Error	t-value	R^2	p-value
High	Intercept	25.53175	0.07022	363.584	0.01225	0.06067
	Slope	-0.01339	0.02564	-0.522		
Medium	Intercept	28.93401	0.11712	247.047	0.02306	0.4787
	Slope	0.03082	0.04277	-0.04277		
Low	Intercept	32.22962	0.219735	146.675	0.0001573	0.9536
	Slope	0.004721	0.080236	0.059		

Table S5.2 ddPCR raw data for value assignment for low, medium, and high concentration samples of PTI.

Level	Sample	Value (cp/µl)		
		Replica 1	Replica 2	Replica 3
Low	U-438	16,18	12,45	11,22
	U-149	15,13	12,14	15,20
	U-791	17,10	7,25	17,03
	U-448	10,17	13,11	13,75
	U-849	13,82	18,41	16,44
Medium	U-704	157,97	143,43	139,66
	U-761	151,11	138,22	148,19
	U-975	146,61	138,61	129,47
	U-690	151,66	148,49	150,29
	U-512	152,91	161,33	131,60
High	U-797	1435,08	1498,56	1445,57
	U-970	1434,86	1372,10	1418,57
	U-295	1367,88	1300,29	1393,73
	U-325	1319,90	1554,06	1643,59
	U-659	1361,35	1362,41	1352,41

Table S5.3 Analysis of variance of results of table S1 data

Level	Mean	Source	Square sum	GF	Mean square	F	P	F table
Low	14.6	Factor	47.228956	4	11.80724	3.1601	0,06374	3,478
		Rep.	37.36338	10	3.736338			
Medium	146	Factor	256.25765	4	64.064414	0.7118	0.60236	3.478
		Rep.	900.05944	10	90.005944			
High	1417	Factor	51483.699	4	12870.924	1.9793	0.17381	3.478
		Rep.	65028.071	10	6502.8071			

As an informative value, RdRp copy number was measured in the three positive samples in one panel in triplicate using RT-ddPCR to check the assigned value in the second fragment of the RGTM 10169 NIST material.

Table S5.4 PTI sample concentrations using the RdRp assay (one panel).

Level	Concentration (copies/µL)	u (%)	U* (%)
Low	14	19.9	41.9
Medium	131	11.0	23.0
High	1217	4.1	8.6

* Expanded uncertainty at k = 2.1 for a 95% confidence level.

TextS6: Proficiency Test Platform Results

Table S6.1 Platforms and reagents information reported by laboratories.

	Manufacturer	Reference	Number
Open platforms	Bio-Rad Laboratories	CFX Touch 96-well	44
	Thermo Fisher Scientific Inc.	Applied Biosystems™ 7500 Real-Time PCR Systems	10
	Roche Diagnostics	Cobas® Z-480	8
	QIAGEN	Rotor-Gene 6000	6
	Thermo Fisher Scientific Inc.	QuantStudio™ 3 Real-Time PCR System	5
	DNA TECHNOLOGY	Dtprime 4	3
	Roche Diagnostics	LightCycler® 480 Instrument II	3
	Thermo Fisher Scientific Inc.	StepOne ™ Real time PCR system	3

	Agilent Technologies, Inc.	AriaMx Real-Time PCR system	2
	Coyote Bioscience Co., Ltd.	FlashDetect™ Mini8 Plus	2
	Thermo Fisher Scientific Inc.	QuantStudio ™ 5 Real time PCR system	2
	Bioer Technology	Line-Gene 9600 Plus RT-PCR System	1
	Sacace Biotechnologies Srl	SaCycler-96 Real time PCR system	1
Closed platforms	Abbott Laboratories	Abbott m2000 RealTime System	7
	Becton, Dickinson and Company	BD MAX™ System	2
	BioFire Diagnostics	BIOFIRE ® FILMARRAY® 2.0	10
	BIONEER CORPORATION	ExiStation™ 48: ExiPrep™ 48, Exicycler™ 96	5
	Cepheid	GeneXpert®	1
	Roche Diagnostics	cobas® 6800 System	2
	Seegene Inc	STARLet	3
	ELITech Group	ELITe InGenius®	1
Commercial kits for open platforms	Seegene Inc	Allplex™ 2019-nCoV assay	16
	Roche Diagnostics	LightMix. ®. SarbecoV E-gene plus	9
	ELITech Group	GeneFinder™ COVID-19 Plus RealAmp Kit	10
	CerTest Biotec S.L	VIASURE SARS-CoV-2 real time PCR detection kit	5

	DaAn Gene Co., Ltd	DaAnGene - Detection Kit for 2019 Novel Coronavirus (2019-nCoV)	3
	Bioeksen R&D Technologies INC.	Bio-Speedy® Direct RT-qPCR SARS-CoV-2	2
	Coyote Bioscience Co., Ltd.	DirectDetect™ SARS-CoV-2 Detection Kit	2
	Co-Diagnostics, Inc.	LOGIX SMART™ Coronavirus Disease 2019 (COVID-19) Kit	2
	BIONEER CORPORATION	AccuPower® SARS-CoV-2 Real-Time RT-PCR Kit	1
	BioFire Diagnostics	BIOFIRE ® Respiratory 2.1 (RP2.1) Panel	1
	-	eDiagnosis - SARS-CoV-2	1
	Siemens Healthcare GmbH ©	FTDTM SARS-CoV-2 Assay	1
	genesig®	Genesig® Real Time PCR	1
	Liferiver Bio-Tech (US) Corp.	Novel Coronavirus (2019-nCoV) Real Time Multiplex RT-PCR Kit	1
	TIB Molbiol Syntheselabor GmbH	Sarbeco E-gene, Tib Molbiol	1
	Seasun Biomaterials Inc	U-TOP™ COVID-19 Detection Kit	1
	Takara Bio Inc	Takara SARS-CoV-2 Direct PCR detection kit	1
	BioFire Diagnostics	BioFire-(RP2.1)	10

Commercial kits for closed platforms	BIONEER CORPORATION	AccuPower® SARS-CoV-2 Real-Time RT-PCR Kit	6
	Abbott Laboratories	Abbot - RealTime Sars CoV-2 Assay	5
	Seegene Inc	Allplex™ 2019-nCoV assay	3
	Becton, Dickinson and Company	BD MAX™ TNA MMK	2
	CerTest Biotec S.L	VIASURE SARS-CoV-2 real time PCR detection kit	2
	Co-Diagnostics, Inc.	LOGIX SMART™ Coronavirus Disease 2019 (COVID-19) Kit	2
	Cepheid®	Xpert® Xpress SARS-CoV-2	1

Text S7: Proficiency Test Results, Control Use Report

From the participating laboratories, 101 reported the use of positive controls. For commercial kits and closed platforms, 72.1% of laboratories used the positive control included in the commercial kit used. For in-house protocols, 12.6% of laboratories used controls that had been developed in-house and 6.3% of laboratories used controls acquired from an external supplier or one that differed from the kit manufacturer (NIST, INS, or manufacturers such as SeraCare, Eurofins, or Vircell; Figure S3 blue bars).

Regarding negative controls, participating laboratories used a range of materials; 46.8% used the negative control included in the commercial kit used, 29.7% used nuclease-free water, 1.8% used controls from an external supplier or one that differed from the kit manufacturer, and 9.0% used no template control (Figure 5, red bars).

Figure S7.1. Types of positive (blue) and negative (red) controls used by laboratories.

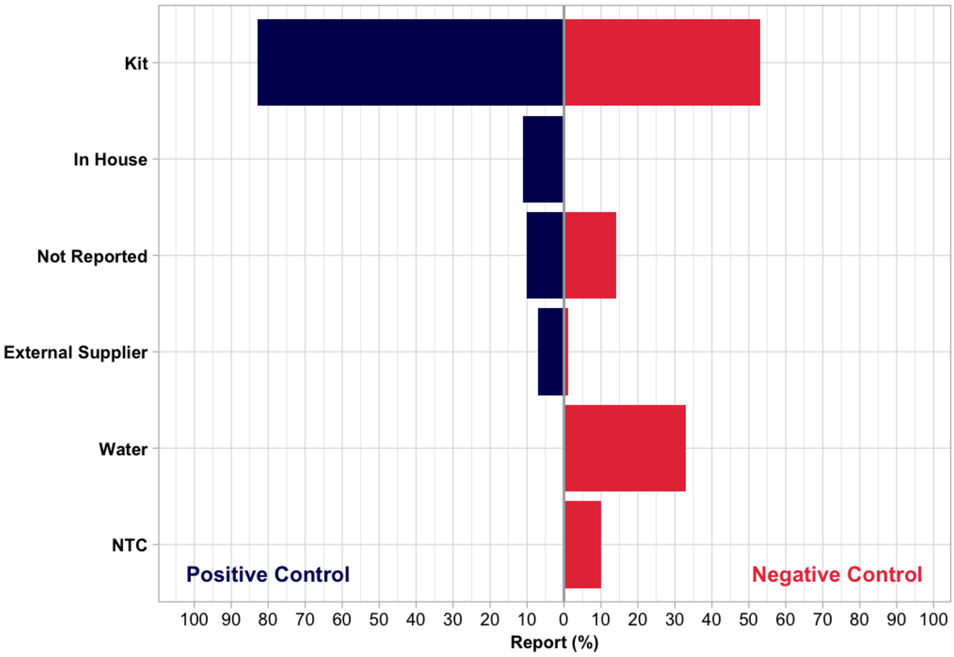

Text S8: Proficiency Test Results, Limit of detection values and PTI volumes reported by laboratories.

The LoD values reported by participating laboratories, based on the information provided in the RT-PCR kits used, values obtained during the method verification, or reported by previous scientific literature, included a wide variety of values and units (copies/µL, copies/mL, copies/reaction, genomic equivalents/mL, µM, ng/µL, and Ct values, among others; Table S9).

Table S8.1. Range and frequency of limit of detection values reported by laboratories.

Reported unit	Reported range	Proportion of reporting laboratories
Copies/reaction*	3.4–100	43%
Copies/mL	4.167–2000	19%
Ct	35–40	16%
Copies/μL	0.33–10	6%
GE/mL	40	2%
ng/μL	1.0×10^{-7}	1%
μM	1.0×10^{-6}	1%
NA	-	12%

*Laboratories reporting only copy numbers or copy number/test were classified as copies/reaction.

Most laboratories used 5 μL of the PTI solution (Figure S4), equivalent to 70 copies per reaction.

Figure S8.1. Volumes of the PTI used for RT-PCR reactions using open platforms.

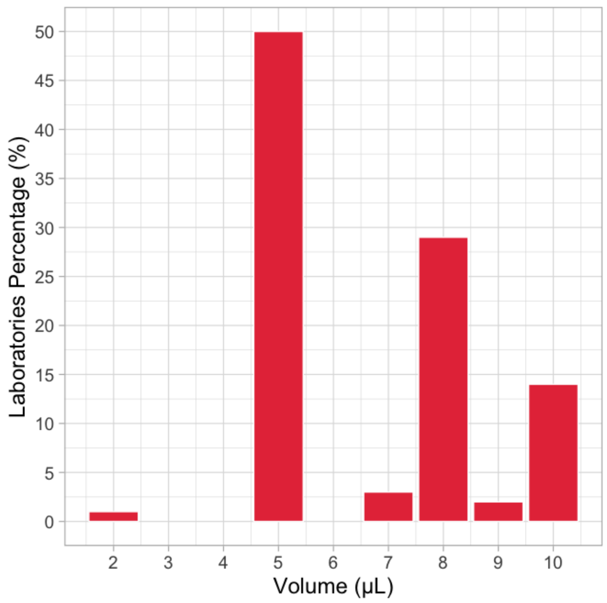

Laboratories reported instrumental response values from one, two, or three replicates depending on the protocol and assay used (Table S10).

Table S8.2. Proportions of laboratories reporting instrumental response values from one, two, or three replicates according to the gene tested.

Gene	One replicate (%)	Two replicates (%)	Three replicates (%)
E	48.9	43.8	32.8
RdRp	22.5	22.9	29.9
N	28.6	33.3	37.3

References

[1] V. M. Corman et al., "Detection of 2019 novel coronavirus (2019-nCoV) by real-time RT-PCR," Eurosurveillance, vol. 25, no. 3, Jan. 2020, doi: 10.2807/1560-7917.ES.2020.25.3.2000045.

[2] National Instituote For Viral Disease Control and Prevention, "Specific primers and probes for detection 2019 novel coronavirus," China CDC. Jan. 21, 2020. Accessed: Jan. 03, 2023. [Online]. Available: https://ivdc.chinacdc.cn/kyjz/202001/t20200121_211337.html

[3] Division of Viral Diseases and Centers for Disease Control and Prevention (CDC), "2019-Novel Coronavirus (2019-nCoV) Real-time rRT-PCR Panel," Atlanta, GA, Jan. 2020. Accessed: Jan. 03, 2023. [Online]. Available: https://www.who.int/docs/default-source/coronaviruse/whoinhouseassays.pdf
